# Human α-defensin 6 (HD6) suppresses CRC proliferation and metastasis through abolished EGF/EGFR signaling pathway

**DOI:** 10.7150/ijms.64850

**Published:** 2022-01-01

**Authors:** Po-Li Wei, Jang-Chun Lin, Chin-Sheng Hung, Precious Takondwa Makondi, Uyanga Batzorig, Tung-Cheng Chang, Chien-Yu Huang, Yu-Jia Chang

**Affiliations:** 1 Division of Colorectal Surgery, Department of Surgery, Taipei Medical University Hospital, Taipei Medical University, Taipei, Taiwan.; 2 Department of Surgery, College of Medicine, School of Medicine, Taipei Medical University, Taipei, Taiwan.; 3 Cancer Research Center and Translational Laboratory, Department of Medical Research, Taipei Medical University Hospital, Taipei Medical University, Taipei, Taiwan.; 4 Graduate Institute of Cancer Biology and Drug Discovery, Taipei Medical University, Taipei, Taiwan.; 5 Department of Radiation Oncology, Shuang Ho Hospital, Taipei Medical University, Taipei, Taiwan.; 6 Department of Radiology, School of Medicine, College of Medicine, Taipei Medical University, Taipei, Taiwan.; 7 Kamuzu Central Hospital - National Cancer Center, Lilongwe, Malawi.; 8 Division of General Surgery, Department of Surgery, Shuang Ho Hospital, Taipei Medical University, Taipei, Taiwan.; 9 Division of Colon and Rectal, Department of Surgery, Shuang Ho Hospital, Taipei Medical University, Taipei, Taiwan.; 10 Graduate Institute of Clinical Medicine, School of Medicine, College of Medicine, Taipei Medical University, Taipei, Taiwan.; 11 Cell Physiology and Molecular Image Research Center, Wan Fang Hospital, Taipei Medical University, Taipei, Taiwan.; 12 Department of Pathology, Wan Fang Hospital, Taipei Medical University, Taipei, Taiwan.

**Keywords:** Colon cancer, defensin6, progression, migration, serpine-1, EGF, EGFR

## Abstract

The incidence of colorectal cancer (CRC) has increased significantly in the past decade. Early diagnosis and new therapeutics are still urgently needed for CRC in clinical practice. Human α-defensin 6 (HD6) plays a defense role against microbes in the gastrointestinal tract. However, the role and mechanism of HD6 in CRC is still unresolved. Specimens from CRC patients with higher HD6 showed better outcomes. Overexpressed HD6 in CRC cells caused a reduction of cell proliferative, migratory, and invasive ability *in vitro* and *in vivo*. HD6-overexpressed caused S phase arrest through changes in cyclin-A and B and CDK2 levels. In addition, serpine-1 may be negatively regulated by HD6 altering the translocation of c-Jun N-terminal kinases (JNK), extracellular regulated protein kinases (ERK), and p38. Higher HD6 and lower serpine-1 levels in CRC patients reflected better outcomes. Finally, we found that HD6 interacts directly with epidermal growth factor receptor (EGFR) by co-immunoprecipitated assay. EGF treatment caused an increase of the level of serpine-1 and pEGFR levels and then increased growth activity in HD6 overexpressing cells. Together, our study shows that HD6 may compete with EGF to bind to EGFR and interrupt cancer progression in CRC. We believe these findings may give new insights for HD6 in CRC therapy.

## Introduction

The incidence of colorectal cancer (CRC) is still in the top five among all cancers worldwide 1. Many patients are diagnosed with already metastatic disease, and there are high rates of resistance and recurrence after treatment 2. This leads to progressive disease with poor overall and disease-free survival 3, 4. Most CRC develops through a multistep process, from benign adenomas to larger and more dysplastic lesions, eventually becoming malignant 5. Currently, the major strategy for early diagnostic CRC were surgery alone or in combination with chemotherapy and radiotherapy. However, surgery is no longer effective for advanced stages that represent 25% of CRCs cases 6. Unfortunately, the rapid evolution of drug resistance and the occurrence of cancer recurrence remains a clinical obstacle for CRC therapy 7. Hence, figuring out other treatment options for CRC, especially for metastatic CRC, is highly needed.

Defensins are 30- to 40- amino acid cationic peptides possessing a conserved structural fold and containing six highly conserved cysteine residues that form three pairs of intramolecular disulfide bonds 8. The disulfide bonds serve to stabilize the molecules as triple-stranded amphiphilic sheet structures 8. Six α-defensins and 31 β-defensins have been identified in humans 9, 10. The human α-defensin family includes human neutrophil peptides 1-4 (HNP 1-4), which are expressed primarily by granulocytes, and human defensin 5 and 6 (HD5 and HD6), which are generated primarily by Paneth cells in the small intestine 11-13. HD6 is involved in the mucosal defense against microbes in the gastrointestinal tract^14^ and found significantly increased in colonic intestinal epithelial cells of ulcerative colitis patients 16. HD6 exhibit significantly high expression in CRC tissues 15, 16. However, the exact role of HD6 in the development of CRC remains elusive.

Using bioinformatics analysis, we demonstrated that a high level of HD6 was correlated with good overall survival (OS), suggesting its involvement in CRC progression. Overexpressed HD6 caused an inhibitory effect on cancer growth, metastasis, and invasion. To explore its mechanisms in CRC, HD6 may compete with the interaction between epidermal growth factor (EGF) and epidermal growth factor receptor (EGFR) and cause a reduction of serpine-1 expression in CRC cells. In-depth understanding of the HD6-associated pathway will provide new insights into the treatment of CRC.

## Materials and Methods

### Chemicals and reagents

Propidium iodide (PI), Tris-HCl, trypan blue, ethylenediaminetetraacetic acid (EDTA), 3-(4,5-dimethylthiazol-2-yl)-2,5-diphenyltetrazolium bromide (MTT), ribonuclease A, and dimethyl sulfoxide (DMSO) were obtained from Sigma Chemical (St. Louis, MO, USA). Anti-HDA6 antibody was purchased from Novus Biologicals, LLC (Centennial, CO, USA). Anti-serpine-1, EGFR, β-catenin, vimentin, Cyclin-dependent kinase 2 (CDK2), fibronectin, Poly (ADP-ribose) polymerase (PARP), and Glyceraldehyde-3-Phosphate Dehydrogenase (GAPDH) antibodies were purchased from Santa Cruz Biotechnology (Santa Cruz, CA, USA). Anti-p38, phospho-p38, c-Jun N-terminal kinases (JNK), phospho-JNK, extracellular regulated protein kinases (ERK), and phospho-ERK antibodies were purchased from Cell Signaling Technology (Danvers, MA, USA). Anti-snail antibody was purchased form GenScript (Piscataway, NJ, USA). Anti-phospho-snail and Cox-2 antibodies were purchased from Abcam (Cambridge, MA, USA). Anti-cyclin A2 and cyclin B1 were purchased from GeneTex, Inc. (Irvine, CA, USA).

### Cell culture

CaCO_2_ human CRC cells were provided by Dr. Wei-Chiao Chang (Pharmacy Department, Taipei Medical University). HCT-116, HT29, and DLD-1 cell lines were purchased from the American Type Culture Collection (ATCC; Manassas, VA, USA). The cells were cultured in RPMI with 10% fetal bovine serum (FBS) in a humidified incubator (37 °C, 5% CO_2_). The cells were either subcultured or used before they reached 80% confluence.

### Bioinformatic data resources

To assess the role of HD6 gene in CRC, the Gene Expression Omnibus (GEO) database (http://www.ncbi.nlm.nih.gov/geo/) 17 was used to identify datasets that contained gene expression data and clinical information of CRC patients. The series matrix files of GSE12945, GSE14333, GSE17536, GSE17537, GSE31595, and GSE41258 datasets were downloaded and this totaled 482 cases. HD6 was identified by the probe set ID 207814_at. The survival profiles were compared on the basis of a high or low expression of the gene, and the overall survival (OS) in months was assessed. To identify if the gene is highly or low expressed, z-scores were calculated in the individual datasets. The z-score was calculated by the formula z = (x-μ)/σ, where x is gene expression value in a specific patient, μ is the average gene expression and σ is the standard deviation of the datasets gene expression. In this case low expression was negative z-score and positive z-score was high expression. Kaplan-Meier survival curves were plotted using SPSS for Macintosh (version 21; IBM Corp., Armonk, NY, USA; www-01.ibm.com), and log rank p-value (<0.05 as significance value) were calculated. The survival plots of serpine-1 identified by probe set 202627_s_at were also determined using the method described above.

### Generation of HD6 overexpression cells

HD6 was overexpressed in DLD-1 and HT29 cells by transfected pCMV6-HD6 plasmid using the Neon® Transfection System (Life Technologies, Grand Island, NY, USA) as previously described 18, 19. Stably transfected cells were selected by antibiotic. The level of HD6 was determined by QPCR and Western blotting.

### Protein extraction preparation and Western blot analysis

The cells were lysed using cell lysis buffer (Sigma-C2978) containing protease inhibitors (Complete Protease Inhibitor Tablets; Boehringer Mannheim, Indianapolis, IN, USA). The proteins (25 μg) in each sample were separated using 10% SDS-PAGE and transferred onto poly-vinylidene fluoride (PVDF) membranes (GE Healthcare). The membranes were blocked and then incubated overnight at 4 °C with a primary antibody against a specific target, and subsequently probed with a horseradish peroxidase-conjugated secondary antibody (1:5000). The signals were visualized with a chemiluminescence reagent (GE Healthcare) and detected using a VersaDoc 5000 (Bio-Rad Laboratories, Hercules, CA, USA).

### Flow cytometry for cell-cycle analysis

Cells (3 × 10^5^) were seeded onto 6-well plates overnight. At specific time intervals, cells were harvested, washed with PBS, fixed in pure methanol, treated with RNase A (at a final concentration of 40 μg/ml), and finally stained with PI (40 μg/ml) for 30 min at room temperature. The stained cells were analyzed using a flow cytometer (BD Biosciences, San Jose, CA, USA), and the DNA content was quantified using Modfit software (Verity Software House, Topsham, ME, USA).

### Immunofluorescence staining

The cells were seeded on glass coverslips overnight and then fixed in 4% paraformaldehyde for 15 min at room temperature (RT). The fixed cells were permeabilized with 0.1% Triton X-100 and incubated with 5% bovine serum albumin (BSA) blocking buffer for 30 min. After being washed with PBS, the cells were incubated overnight at 4 °C with a rabbit anti-HD6 antibody and then for 1 h RT with CF^TM^ 488-labeled anti-rabbit secondary antibody (Sigma Chemical Co.). The coverslips were then mounted with vectashield containing DAPI (Vector Laboratories), and the cells were examined by fluorescence microscopy (Olympus America, Inc.).

### Co-immunoprecipitation

After exposure to EGF treatment (500 ng/ml) or not, cells were washed and lysed. Then 200 µg of protein extract was incubated with 2 μg of anti-EGFR antibodies and 30 μL of Protein G beads (Santa Cruz Biotechnology, Milan, Italy) overnight to allow complex formation. Samples were then centrifuged at 6000 rpm for 3 min to pellet beads. Pellets were washed 4 times with immunoprecipitation buffer, centrifuged at 6000 rpm for 3 min, and boiled with SDS-PAGE loading buffer. Proteins were then resolved using SDS-PAGE, transferred to PVDF membrane, and probed with primary antibodies. Western blot analysis and enhanced chemiluminescence (ECL) detection were performed as described above.

### Evaluation of cell proliferation and migration using xCELLigence biosensor system

Experiments were performed using a real-time cell analysis dual-plate (RTCA DP) instrument (ACEA BioSciences, Inc., San Diego, CA, USA) that was placed in a 5% CO_2_ humidified incubator maintained at 37 °C. Growth curves were constructed using 16-well plates (E-Plate 16, ACEA BioSciences, Inc., San Diego, CA, USA). Cells were seeded in an E-Plate 16 at 10,000 cells/well in FBS-containing medium. The plates were then monitored once every 30 s for 4 h and once every half hour thereafter. The data were analyzed using RTCA software 1.2 (supplied with the instrument) 20, 21.

### Evaluation of cell proliferation using SRB assay

Cell proliferation was also assessed by sulforhodamine B (SRB) assay, where about 4 × 10^4^ vector control and HD6 overexpressing cells were seeded into 96-well plates (Falcon, Germany) and incubated for 48 h. The cells were then washed with PBS, fixed with 10% trichloracetic acid (TCA), and stained with SRB dye, after which the dye was dissolved in 10 mM Tris and the absorbance was measured with an ELISA reader (SLT-rainbow) at 540 nm.

### *In vivo* tumor xenograft experiments

All mouse experiments were performed in strict accordance with the regulations of the Institutional Animal Care and Use Committee (IACUC), Taipei Medical University. Male nude mice (5 weeks old) were used for the *in vivo* experimental model. Control and HD6 overexpressed DLD-1 cells were suspended in PBS to a final cell density of 1 × 10^7^ cells/mL. A volume of 0.1 mL of the cell suspension was injected subcutaneously into the bilateral flanks of each mouse. Tumor dimensions and body weights were recorded twice per week. Tumor volumes were calculated using the equation (L × w^2^)/2, where L and w are the larger and smaller tumor dimensions, respectively 22. After 5 weeks, the mice were sacrificed, and all tumors were excised and weighed. Half of the excised tumor tissue was fixed in 10% formalin and embedded in paraffin for immunohistochemical staining; the other half was snap-frozen in liquid nitrogen for further evaluation.

### Transwell migration assay and invasion assay

*In vitro* cell migration and invasion were examined using the BD Falcon cell culture insert (BD Biosciences) and the BD BioCoat™ Matrigel Invasion Chamber (BD Biosciences), respectively. Aliquots of 1 × 10^5^ cells expressing scrambled control or overexpressing HDA6 were suspended in 500 μL of serum-free RPMI and seeded into the upper compartment of each chamber. The lower compartments were filled with 1 mL of RPMI with 10% FBS. After incubation for 48 h at 37 °C in 5% CO_2_, nonmigrating and noninvading cells were removed from the upper surface of the membrane by scrubbing. The cells on the reverse side were stained with 0.1% crystal violet, and migrating and invading cells were counted under a microscope at 100× magnification.

### Wound-healing assay

The wound-healing assay was performed using 5 × 10^5^ control or HD6 overexpressing cells in 70 μL of dimethyl sulfoxide (DMEM) containing 10% fetal calf serum seeded into ibidi cell culture inserts (ibidi GmbH, Munich, Germany) in 35 mm dishes and incubated at 37 °C in 5% CO_2_. After 24 h, the culture inserts were removed and added to the media. The cell-free gap was monitored under time-lapse microscopy (Lumascope 500× video microscope). The gap was analyzed with ImageJ software.

### Statistical analysis

All experiments were repeated a minimum of 3 times. All data collected from real-time RT-PCR and cell proliferation experiments are expressed as means ± SD. The data presented in some figures are derived from a representative experiment that was quantitatively similar to the replicate experiments. When 2 groups of datasets were compared, statistical significance was determined using a 2-tailed Student's *t* test. Asterisks in the figures indicate significant differences between the indicated experimental groups and the corresponding control conditions (P < 0.05; see figure legends).

## Results

### HD6 expression is correlated with CRC patient outcome

**Figure 1:**
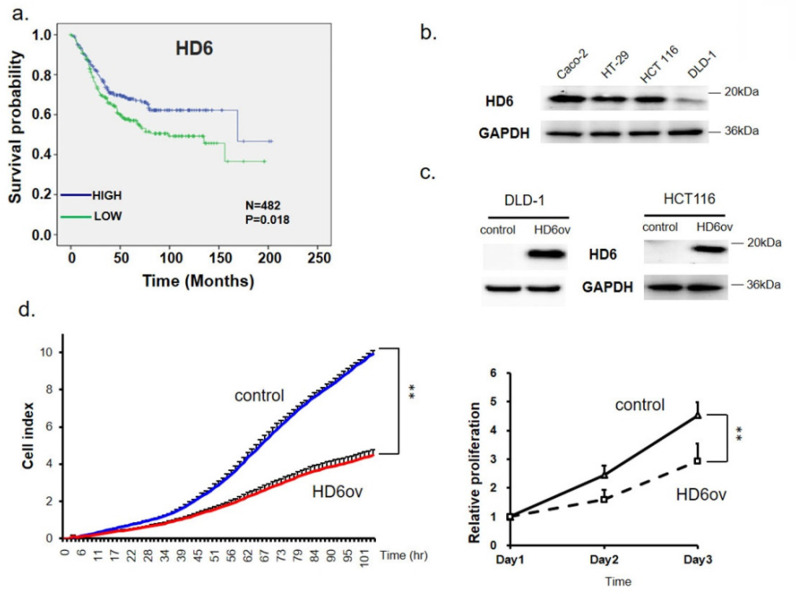
Human defensin alpha 6 (HD6) levels correlated to overall survival and growth activity in colorectal cancer (CRC). (**a**) Kaplan-Meier survival curve presenting the prognostic relationship between high and low expression of HD6 gene. HD6 expression was dichotomized by z-score value and P-values were calculated using the log-rank statistic; patient number (N) = 482. (**b**) HD6 expression in different CRC cell lines determined by Western blot; GAPDH used as internal control. (**c**) Confirmation of HD6 overexpression in DLD-1 and HCT116 cells. (**d**) Proliferation of DLD-1 vector control and overexpressed HD6 (HD6ov) cells as determined by xCELLigence (left panel) and sulforhodamine B (SRB) assay (right panel). ** P < 0.01.

First, a bioinformatics approach was applied to explore the role of HD6 in OS of CRC patients in six pooled GEO datasets. The probe set used to identify HD6 was 207814_at, and there were a total of 482 patients. The plotted Kaplan-Meier survival curve demonstrated that high HD6 expression was associated with favorable OS (Fig. 1a). This result suggests that HD6 may have a role in CRC progression.

### Overexpressed HD6 in CRC cells suppresses cell growth

The expression pattern of HD6 in human CRC cell lines (CaCO_2_, HT29, HCT-116, and DLD-1) was assessed using Western blot. As shown in Fig. 1b, CaCO_2_ highly expressed HD6 more than other cells, followed by HT29 and HCT-116 cells, with DLD-1 expressing the least HD6. Then, we overexpressed HD6 in HCT-116 and DLD-1 cells (Fig. 1c). Cell proliferation was monitored by biosensor or SRB assay. We found that HD6 overexpressed (HD6_ov_) DLD-1 and HCT-116 cells had lower proliferative activity compared with vector control cells (Fig. 1d), indicating that HD6 may inhibit CRC proliferation.

### Overexpressed HD6 caused S phase arrest

**Figure 2:**
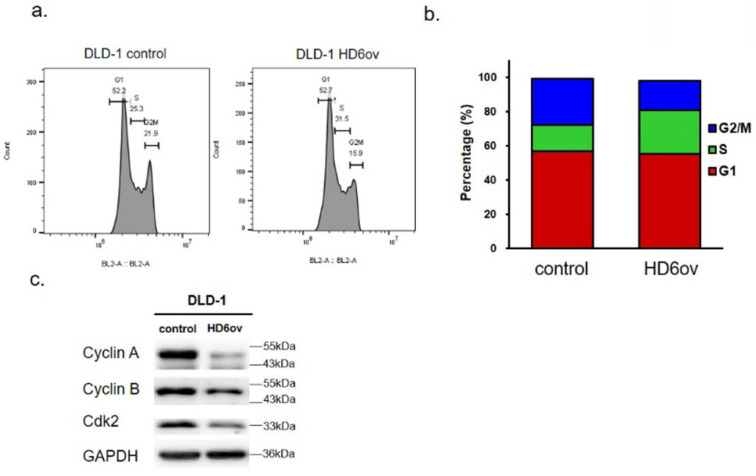
Cell cycle distribution analysis of control and HD6ov cells. (**a**) Flow cytometric cell cycle analysis performed by staining DNA with propidium iodide (PI). (**b**) Cell cycle distribution (G1, S, G2/M). (**c**) Protein expression levels of cyclin A2, cyclin B1, and CDK2 examined by Western blot.

To understand the mechanism of HD6 in cell proliferation, the cell cycle distribution was analyzed using flow cytometry in control and HD6ov DLD-l cells. As shown in Fig. 2ab, the S phase cell population in HD6 overexpressing cells was increased, and there was a decrease in the G_2_-M phase population. There was no significant change in the G_1_ phase between control and HD6ov cells. Further, the levels of cell cycle regulatory proteins were assessed, and it was found that overexpressed HD6 led to decreased expression levels of cyclin-A and B and Cdk2 (Fig. 2c). These results indicate that HD6 may mediate cell proliferation through regulation of the S phase cell cycle transition.

### Overexpressed HD6 reduces cancer progression in xenograft mice models

**Figure 3:**
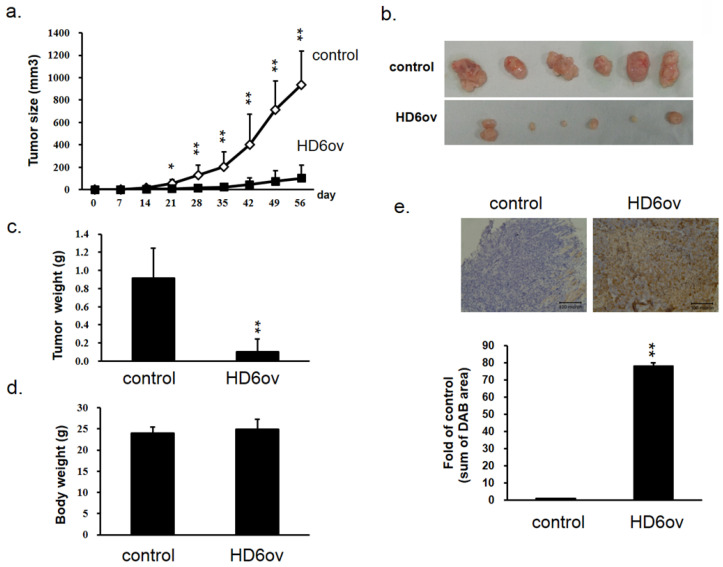
HD6 suppresses cancer progression in xenograft models. (**a**) Tumor volume in control and HD6ov groups was measured. (**b**) Outlooks for control and HD6ov groups after animal sacrifice at day 28. (**c**) Tumor weights. (**d**) Body weights of control and HD6ov groups. (**e**) HD6 levels in control and HD6ov tumors were monitored by immunohistochemical (IHC) staining. * P < 0.05, ** P < 0.01.

To further confirm the *in vitro* findings, a xenograft model was used to evaluate the role of HD6 in CRC progression. The tumor growth rate in mice injected with HD6 overexpressing cells was significantly decreased compared with that in mice injected with control cells (Fig. 3a), and the HD6ov tumors were smaller than the control tumors (Fig. 3b). Although the tumor weights in the HD6ov group were reduced dramatically (Fig. 3c), there was no significant difference in the body weights between HD6ov and control groups (Fig. 3d). Immunohistochemical (IHC) staining in tumor tissues demonstrated that the tumors in the HD6ov group highly expressed HD6 more than the control tumors (Fig. 3e). These results indicate that HD6 influences CRC progression *in vivo*.

### Overexpression of HD6 inhibits cell migration and invasive ability

**Figure 4:**
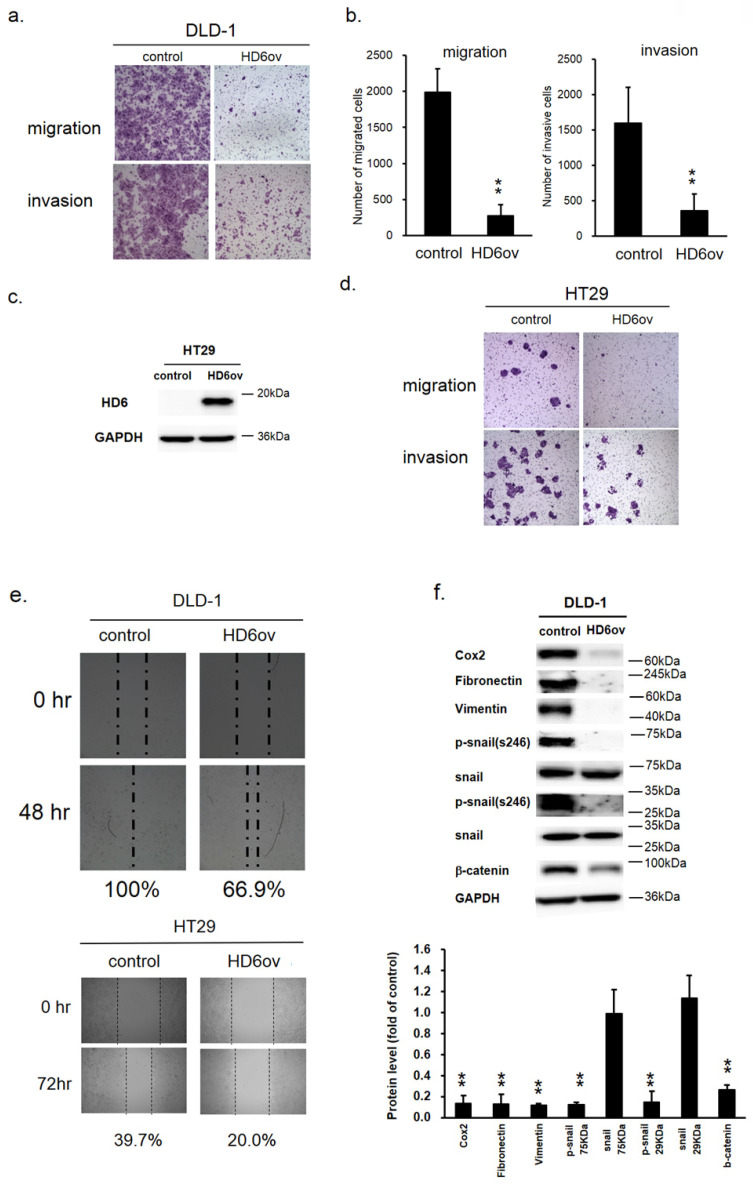
HD6 overexpression suppresses metastatic ability of CRC cells. (**a,b**) Migratory and invasive ability of control and HD6ov DLD-1 cells as determined by transwell migration and invasion. (**c,d**) Overexpressed HD6 cells reduced migratory and invasive ability in HT29 cells. (**e**) Wound healing ability was reduced in HD6ov CRC cells (DLD-1 or HT29) compared with control cells. (**f**) Levels of Cox-2, fibronectin, vimentin, p-snail, snail, and beta-catenin were determined by Western blot. ** P < 0.01.

Biosensor, transwell migration, and invasion assays were performed to monitor the effect of HD6 on the migratory and invasive ability in CRC cells. As shown in Fig. 4a,b, the migratory and invasive ability was reduced significantly in DLD-1 by overexpressed HD6 (Fig. 4a,b). The quantitative results of transwell migration and invasion assays indicate that the number of migrated and invasive cells was reduced by over 50% in HD6 overexpressing cells compared with control cells (Fig. 4b). Similar results were found in HT29 cells (Fig. 4c,d). In the wound-healing migration assay, we found that the wound gap in control DLD-1 cells went from 100% to 0% after 48 h incubation, but in HD6ov cells remained at 33% after 48 h (Fig. 4e). A similar trend was found in HT29 cells. After 72 h, the wound gap area for control and HD6ov cells remained approximately 60.3% and 80%, respectively. These results indicate that overexpressed HD6 may inhibit the migratory and invasive ability in CRC cells.

### Overexpression of HD6 alters the expression patterns of EMT biomarkers

The epithelial-mesenchymal transition (EMT) is a crucial process in the carcinogenesis of different cancers. Thus, to understand whether the suppression effect of HD6 on migratory and invasive ability may be through modulation of EMT, the expression levels of the EMT markers Cox2, fibronectin, vimentin, phospho-snail, and beta-catenin were examined by Western blotting. As shown in Fig. 4f, the expression levels of all of these markers were reduced dramatically in HD6ov cells. These results suggest that HD6 may mediate cancer metastasis through altering the EMT pathway.

### Overexpressed HD6 suppresses serpine-1 expression

**Figure 5:**
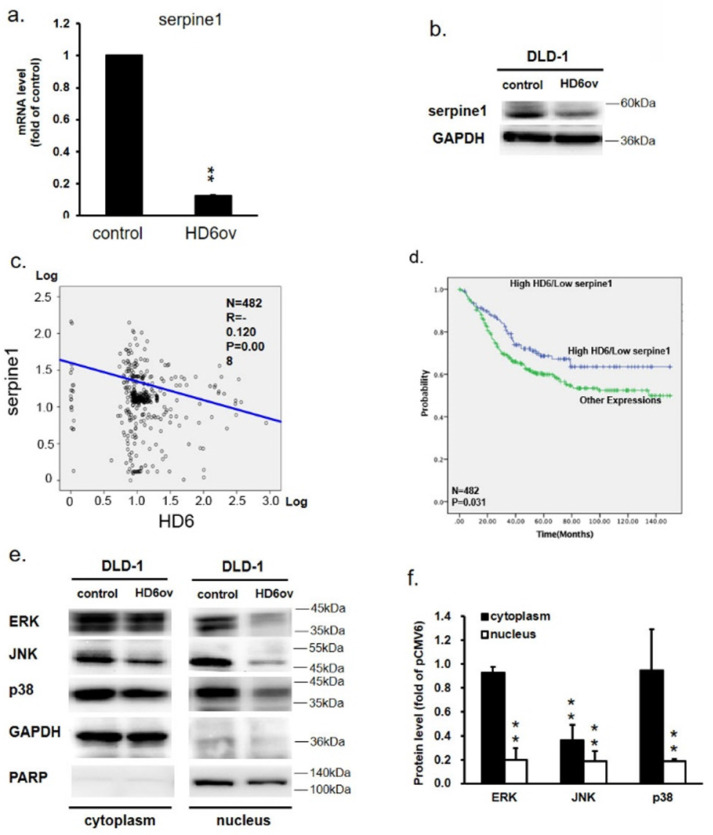
HD6 suppresses serpine-1 expression and is associated with nuclear translocation of MAPK pathway genes (**a,b**) Serpine-1 expression in control and HD6ov DLD-1 cells determined by QPCR and Western blot. (**c**) Expression correlation between serpine-1 and HD6 in pooled Genetic Expression Omnibus (GEO) datasets; patient number (N) = 482; R, Pearson's correlation coefficient; P, P-value. (**d**) Kaplan-Meier survival curve representing prognostic relationship between high HD6 and low serpine-1 compared to other expressions; HD6 and serpine-1 expression was dichotomized by median value and P-values were calculated using the log-rank statistic; patient number (N) = 482. (**e,f**) Levels of ERK, JNK, and p38 in cytoplasm and nucleus determined by Western blot. ** *P* < 0.01.

To further assess the downstream target gene for HD6, we used Nanostrip to analyze the gene expression patterns in control and HD6ov DLD-1 cells. We found that the level of serpine-1 was reduced dramatically by overexpressed HD6 (data not shown). We then conducted QPCR and Western blotting and found that the level of serpine-1 was decreased dramatically at transcriptional and translational levels by overexpressed HD6 in DLD-1 cells (Fig. 5a,b). Then we further confirmed the expression correlation between HD6 and serpine-1 in pooled GEO datasets that had expression patterns of these genes. As shown in Fig. 5c, there was a negative correlation between the expression levels of HD6 and serpine-1. Then we explored the role of high HD6 and low serpine1 in CRC patient outcomes, and as shown in Fig. 5c, high HD6/low serpine-1 showed better survival outcomes in CRC patients. These results indicate that HD6 may modulate the expression of serpine-1 to regulate CRC progression.

### Overexpression of HD6 reduces ERK, JNK, and p38 nuclear translocation

To further dissect how HD6 influences cell proliferation and metastatic ability in CRC, the status of ERK, JNK, and p38 expression in control and HD6ov DLD-1 cells was assessed. Lower ERK, phospho-ERK, phospho-JNK, p38, and phospho-p38 levels were found in HD6ov cells (Fig. 5d). Further, the expression levels of these proteins were also assessed in cytoplasm and nucleus locations. As shown in Fig. 5e, it was found that there was a significant difference in expression of ERK and p38 in the cytoplasm between control and HD6ov cells, but the JNK level was reduced significantly in HD6ov cells. In the nucleus, the amounts of ERK, JNK, and p38 were dramatically reduced in HD6 cells (Fig. 5f). These results indicate that overexpressed HD6 may suppress the translocation of ERK, JNK, and p38 into the nucleus.

### HD6 interacts with EGFR to interfere with the EGF/EGFR pathway in CRC cells

**Figure 6:**
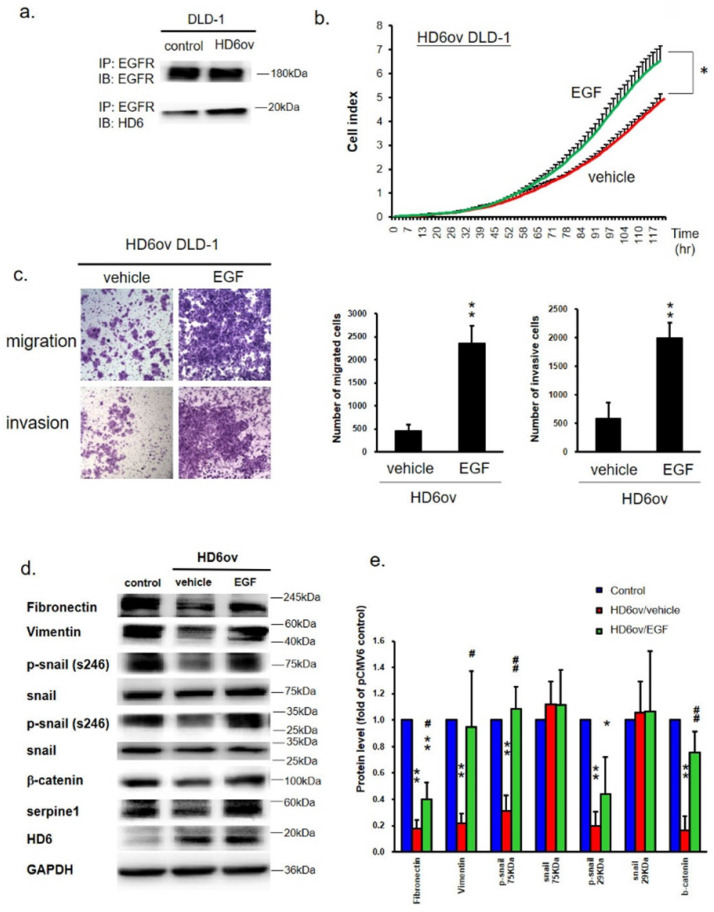
HD6 binds to EGFR to block the effects of EGF/EGFR in CRC. (**a**) Co-immunoprecipitation Western blot assay was performed to see the interaction between HD6 and EGFR. (**b**) Growth activity in HD6ov DLD-1 cells after treatment with vehicle or EGF was determined by the xCELLigence system. (**c**) Migratory and invasion ability of HD6ov DLD-1 treated with vehicle or EGF determined by transwell migration and invasive system. (**d,e**) Levels of fibronectin, vimentin, p-snail, snail, beta-catenin, serpine-1, and HD6 in control DLD-1 and HD6ov cells treated with EGF were determined by Western blot. * *P* < 0.05, ** *P* < 0.01.

It is well known that EGFR plays an important role in CRC progression 23, and EGF/EGFR is also said to go through activation of ERK, JUN, and p38 to modulate CRC progression 24. We propose that HD6 may interact with EGFR to abolish EGF/EGFR activation. Co-immunoprecipitation (co-IP) assay was performed to see whether HD6 interacts with EGFR. As shown in Fig. 6a, the sample was pulled down by anti-EGFR antibody, and detected by anti-EGFR or HD6 antibody. It was found that HD6 could be detected after pull-down by EGFR antibody in both control and HD6ov cells. This result indicates that HD6 may interact with EGFR.

### EGF treatment reverses the suppression effect of HD6

To explore the role of HD6 in the EGF/EGFR pathway, HD6ov cells were treated with different amounts of EGF or vehicle, and then the proliferation, migratory, and invasive abilities were determined. EGF treatment increased the proliferation, migratory, and invasive abilities of HD6 cells as compared to vehicle treatment (Fig. 6b,c). Further, the status of EMT biomarkers (fibronectin, vimentin, snail, and beta-catenin) was assessed by Western blot. EGF exposure caused changed expression patterns of these EMT biomarkers (Fig. 6d,e). The levels of fibronectin, vimentin, p-snail, and beta-catenin increased after EGF treatment (Fig. 6d,e) which is consistent with the increased migratory and invasive ability. Furthermore, the level of serpine-1 was also restored after EGF treatment (Fig. 6d,e). However, EGF treatment did not influence the levels of HD6 and Cox-2. These results suggest that overexpressed HD6 may interfere with the EGF interaction with EGFR to inhibit CRC progression.

### HD6 competes with the EGF interaction with EGFR

**Figure 7:**
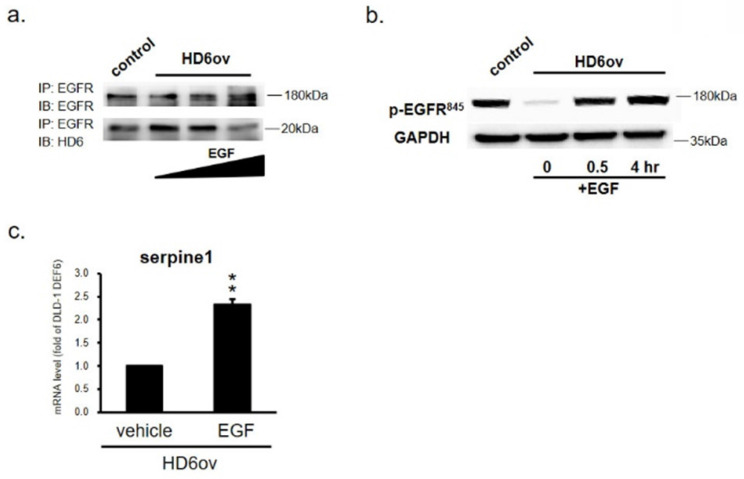
EGF treatment reverses the suppressive role of HD6. (**a**) EGFR and HD6 expression in DLD-1 vector controls and HD6 overexpressing cells treated with vehicle or 500 ng/ml EGF. (**b**) Levels of p-EGFR in DLD-1 vector control and DEFA6 overexpressed cells treated with vehicle or EGF determined by Western blot. (**c**) RNA levels of serpine-1 were examined by qPCR following EGF treatment. ** *P* < 0.01.

In order to further confirm that the inhibitory effect of HD6 is by competing with EGF bound to EGFR, HD6ov cells were exposed to EGF, and then the samples were harvested at different time intervals to perform co-IP assay. As shown in Fig. 7a, it was found that the HD6-EGFR protein interaction decreased with EGF treatment, indicating that EGF may compete with the interaction between HD6 and EGFR. To further examine whether HD6 competes with the EGF interaction with EGFR and regulates the mitogen-activated protein kinase (MAPK) pathway, the expression status of EGFR tyrosine 845 phosphorylation in HD6 overexpressing cells with or without EGF treatment was assessed by Western blot. As shown in Fig. 7b, the level of phospho-EGFR(Tyr845) increased after EGF treatment in HD6ov cells. Moreover, the transcriptional activity of seprpine1 was reversed following EGF in HD6ov cells (Fig. 7c).

## Discussion

**Figure 8:**
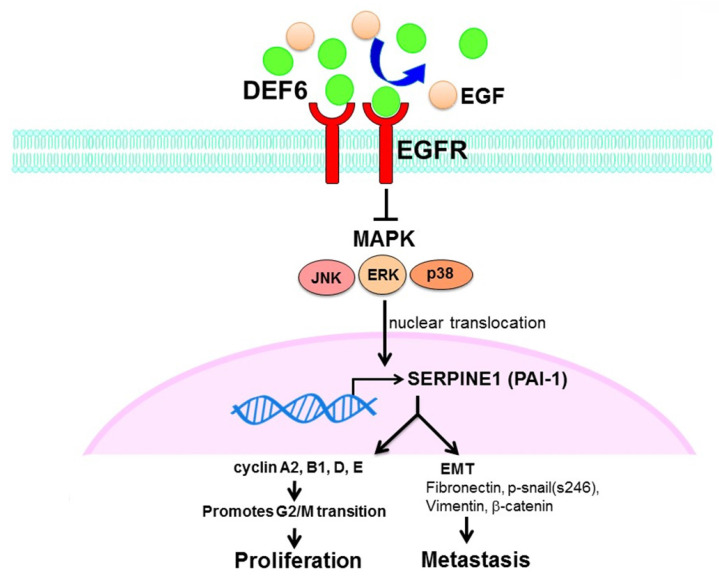
Schematic plot summarizing regulation of HD6 in colorectal cancer, showing how HD6 may compete with EGF binding to EGFR to inhibit the MAPK pathway, resulting in inhibition of proliferation and metastasis.

Patient outcomes in advanced CRC remain dismal, and it is urgent to find new strategies for therapy. Our analysis of bioinformatics databases showed that high expression of human HD6 was associated with favorable survival in CRC. Furthermore, *in vitro* and *in vivo* experimental data show that overexpressed HD6 caused inhibition of cell proliferation, and migration. HD6ov inhibits level of serpine-1, a malignancy biomarker, and high HD6/low serpine-1 was associated with better survival outcome in CRCs. We further demonstrated that HD6 directly interacts with EGFR and interferes with its downstream signal transduction, and these suppressive effects can be reversed with EGF treatment (Fig. 8). Our results suggest that HD6 exerts its modulatory effect on CRC through the EGF/EGFR pathway. These findings demonstrate the comprehensive therapeutic potential of HD6 in CRC.

HD6 is highly expressed in normal mucosa, adenoma, and in colon cancer. The increase in adenoma is 100-fold higher than normal colon tissue 16. Andreu et al. reported elevated expression of HD6 mRNA as well as the known proliferation markers Myc and cyclin D in the initiation of CRC 25. Increased expression of HD6 may indicate a tumor-suppression mechanism or antitumor immunity against carcinogenesis 15. We further demonstrate inhibited proliferative ability in overexpressed HD6 CRC cells. The suppressive effect was mediated through S-phase arrest, as evidenced by the reduced expression of cell cycle regulatory proteins. All our experimental findings attest to the suppressive effect of HD6 on CRC cells.

The MAPK pathways are known to regulate multiple cell functions such as proliferation, differentiation, migration, and apoptosis 26. ERK1/2, JNKs, and p38 are the three of the major MAPK pathways 26, 27. They are mainly activated by growth factors or cytokines and regulate different kinds of cell stresses and are also known to be involved in autoimmunity 26, 27. Deregulation of MAPK signaling pathways is strongly linked to the development and aggressiveness of CRC 26, 28, 29. Our data showed that HD6 overexpression regulating CRC progression by inhibition of the cell cycle and blocks nuclear translocation of the MAPK pathway. Further study is indicated to unmask the inhibitory effect of HD6 in CRCs, and it may offer alternatives in targeting treatments for CRC.

Serpine-1, also known as PAI-1, is the main regulator of the plasminogen activator system, which regulates the formation of plasmin that is associated with degradation of extracellular matrix and cancer cell invasion, metastasis, and apoptosis 30. Enhanced serpine-1 expression is related to increased tumor invasiveness and aggressiveness 31. It has also been known to be a poor prognostic factor in prostate, gastric, colorectal, and head and neck cancers 32. Expression of serpine-1 is upregulated and high serpine-1 mRNA levels are associated with microsatellite instability and high tumor grading in CRCs 31. Chen et al. reported that expression of serpine-1 was increased in CRC patients with liver metastasis and that blockade of serpine-1 expression decreased the number of liver metastases in a nude mouse model 21. Previous study showed that a high preoperative plasma concentration of serpine-1 was associated with shorter survival in patients with CRC 33. Through a bioinformatics approach, Liang et al. reported that expression of serpine-1 was negatively correlated with overall survival in CRC patients, possibly by interacting with vascular endothelial growth factor A in addition to plasminogen activators 34. In our study, we observed that the level of serpine-1 decreased with overexpression of HD6 in DLD-1 cells. Furthermore, we demonstrated that high HD6 and low serpine-1 levels were associated with better survival in CRC patients. These finding may further explain the inhibitory effect of HD6 on CRC cell migration and invasion.

EGFR inhibitors, such as cetuximab, clinically shown benefit in treatment of metastatic CRCs 35, whether used as single agents or in combination with chemotherapy 36. However, resistance to EGFR inhibitors occurs and leads to treatment failure. The results of our study indicate that HD6 exerts an inhibitory effect on the EGF/EGFR pathway at multiple levels. At the receptor level, HD6 competes with EGF for the binding of EGFR. Whether this effect will be synergistic with or antagonistic to other EGFR inhibitors remains to be determined. However, as 40-60% of patients with KRAS wild-type tumors benefit from EGFR inhibition 35, the role of HD6 in these patients merits further investigation. Our results also show a suppressive effect of HD6 on the progression of CRC in a xenotransplantation model and on EMT biomarkers in CRC cell lines, and these effects could be reversed with EGF treatment. Moreover, our study also demonstrates that HD6 suppresses the translocation of downstream effector proteins ERK, JNK, and p38 into the nucleus. Our results suggest that the anticancer effect of HD6 is closely related to the EGF/EGFR pathway and could potentially augment the effects of current anti-EGFR therapy. However, the role of HD6 in the therapeutic efficacy of EGFR inhibitors is worthy to further dissect it. In addition, the role of HD6 in T-cell mediated immune response in cancer therapeutic may be the important issue worth to go further study.

Our study demonstrates that high HD6 expression correlated with favorable survival in CRC patients. We demonstrated the HD6ov inhibit CRC cells in EGF/EGFR pathway signals and alters the expression patterns of EMT biomarkers. The role of HD6 in other signal transduction pathways, such as interaction with serpine-1, should be a target of future exploration. The effect and potential of HD6 treatment in cancer therapy should be assessed comprehensively in future studies. The potential therapeutic role of HD6 in CRCs demands further experiments to clarify.

## Abbreviations

CRC: colorectal cancer
HD6: human α-defensin 6
EGFR: epidermal growth factor receptor
